# How Protein Depletion Balances Thrombosis and Bleeding Risk in the Context of Platelet’s Activatory and Negative Signaling

**DOI:** 10.3390/ijms251810000

**Published:** 2024-09-17

**Authors:** Hector Montecino-Garrido, Andrés Trostchansky, Yolanda Espinosa-Parrilla, Iván Palomo, Eduardo Fuentes

**Affiliations:** 1 Centro de Estudios en Alimentos Procesados (CEAP), ANID-Regional, Gore Maule R0912001, Talca 3480094, Chile; hector.montecino@utalca.cl; 2 Departamento de Bioquímica and Centro de Investigaciones Biomédicas (CEINBIO), Facultad de Medicina, Universidad de la República, Montevideo 11800, Uruguay; trocha@fmed.edu.uy; 3 Interuniversity Center for Healthy Aging (CIES), Centro Asistencial, Docente e Investigación—CADI-UMAG, Escuela de Medicina, Universidad de Magallanes, Punta Arenas 6210427, Chile; yolanda.espinosa@umag.cl; 4 Thrombosis and Healthy Aging Research Center, Interuniversity Center for Healthy Aging (CIES), Interuniversity Network of Healthy Aging in Latin America and Caribbean (RIES-LAC), Medical Technology School, Department of Clinical Biochemistry and Immunohematology, Faculty of Health Sciences, Universidad de Talca, Talca 3480094, Chile

**Keywords:** platelets, signaling, inhibitory, activatory, bleeding

## Abstract

Platelets are small cell fragments that play a crucial role in hemostasis, requiring fast response times and fine signaling pathway regulation. For this regulation, platelets require a balance between two pathway types: the activatory and negative signaling pathways. Activatory signaling mediators are positive responses that enhance stimuli initiated by a receptor in the platelet membrane. Negative signaling regulates and controls the responses downstream of the same receptors to roll back or even avoid spontaneous thrombotic events. Several blood-related pathologies can be observed when these processes are unregulated, such as massive bleeding in activatory signaling inhibition or thrombotic events for negative signaling inhibition. The study of each protein and metabolite in isolation does not help to understand the role of the protein or how it can be contrasted; however, understanding the balance between active and negative signaling could help develop effective therapies to prevent thrombotic events and bleeding disorders.

## 1. Introduction

When megakaryocytes in the bone marrow are fragmented, small anucleated cells called platelets are produced [1,2,3]. Platelets are essential for hemostasis following an injury because they aggregate to form a plug that seals damaged blood vessels [4]. Platelet activation is required to produce this aggregate, which binds different agonists, such as collagen and thrombin, to platelet receptors. This initiates signaling events that result in platelet morphology changes, granule release, and activation of integrin αIIbβ3, also referred to as the fibrinogen receptor.

Despite their size, platelets have a complex structure necessary for a measured hemostatic response, including receptors intended to receive signals from the extracellular medium [5], membrane proteins used as bridges for adhesion and aggregation [6], and granules that store prothrombotic molecules [2], including clotting factors and vasoactive substances released upon platelet activation, to promote clot formation, vessel constriction, and neutrophil recruitment [7]. Additionally, platelets undergo spreading and cytoskeletal rearrangement [8], allowing them to spread and attach to other cells, such as endothelial cells and other platelets, forming stable aggregates contributing to hemostasis [9].

When clotting becomes excessive and uncontrollable, it can lead to thrombosis, a condition where blood clots block blood vessels, potentially causing severe damage to organs by cutting off their oxygen supply [10]. This can have deadly consequences due to hypoxia, where the lack of oxygen delivery is critical [11,12].

On the other hand, if platelet activation is too low, it can cause excessive bleeding, which is particularly dangerous during major surgeries, trauma, or for patients with bleeding disorders like hemophilia [13]. In these cases, the inability to form stable clots at sites of injury can lead to prolonged bleeding and require urgent medical intervention. This can hint at underlying health issues (as detailed in Table 1). A range of conditions including inflammatory diseases, nephrotic syndrome, autoimmune disorders, lung diseases, infections, and cancer can show up with symptoms related to clotting. In these situations, antiplatelet therapy is often used to adjust how certain proteins behave, helping to prevent problems caused by excessive clotting [14,15].

But it is not perfect, as the recurrence of strokes and bleeding events shows the limitations of existing therapies, such as resistance, side effects, and failure to address patients’ specific needs. Advances in platelet biology and drug discovery technologies offer opportunities for the development of effective and targeted therapies but maintaining a careful balance in platelet function is crucial for proper blood clotting without veering into harmful clotting or bleeding. This balance highlights the need to understand the molecular pathways that control platelet activity, which could lead to new and targeted treatments.

## 2. A Simple Overview of the Activation Process

The strength of the activation process and the severity of clotting are influenced by platelet responsiveness to various stimuli, wherein each agonist triggers activation using different receptors. For example, collagen binds to integrins like α2β1, leading to the activation of Src family kinases.

Several agonists can trigger the activation of G protein-coupled receptors, such as thromboxane A2 (TXA2), which binds to the TP receptor; serotonin, which binds to the 5-HT2A receptor; ADP, which binds to the P2Y1 receptor; or thrombin, which generates peptides by cleaving and activating the receptors PAR1 and PAR4.

The other type of activation is induced by the depletion of cAMP, induced by Gi-mediated inhibition of the enzyme adenylate cyclase, which ADP could trigger in P2Y12 and Epinephrine binding to α2-adrenergic receptors. Additional agonists, such as von Willebrand factor (vWF), immune complexes, and specific inflammatory mediators, can activate platelets through receptor-mediated signaling pathways.

This, in turn, depends on the activity of internal cellular proteins [86]. Several agonists can trigger platelet activation and aggregation, as converging pathways induce the activation of phospholipase C (PLC), which hydrolyzes phosphatidylinositol 4,5-bisphosphate (PIP2) to generate inositol trisphosphate production (IP3) and diacylglycerol (DAG), the first one IP3 mobilizes calcium from internal stores, and promotes the activation of downstream targets, such as integrins; while DAG activates protein kinase C (PKC) inducing the phosphorylation of other proteins linked to the integrins on the platelet surface, such as talin, vinculin, and paxillin. All of this allows for rearranging the cytoskeleton and leads to shape change, granule secretion, and platelet aggregation.

## 3. Balance in the Pathways

**Figure 1:**
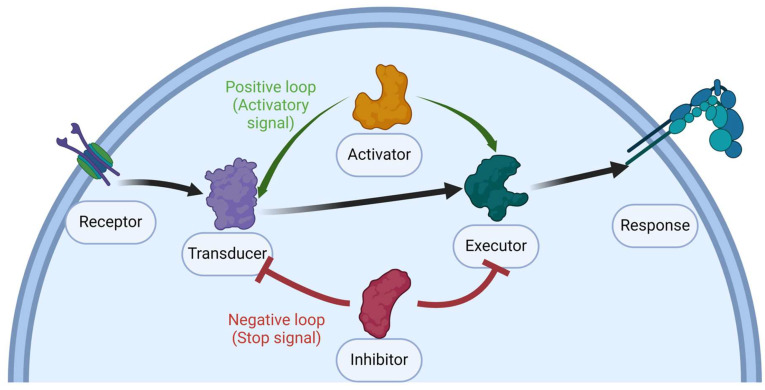
Diagram of Platelet signaling. The illustration represents a cellular signaling pathway, from a receptor’s reception by a signal, its transmission and amplification via a transducer, and the subsequent execution by an effector. Activators and inhibitors modulate the intensity of the signal, while the system’s equilibrium is preserved through positive and negative feedback mechanisms, inducing or reducing the platelet aggregation.

This raises the question of whether platelet inhibition induces bleeding or thrombosis. The answer is that defective or depleted proteins in the regulatory or negative signaling pathway can increase thrombus size, stability, and formation time and diminish bleeding time in vivo [87,88], causing thromboembolic events [89]. Removing active proteins can decrease platelet function and increase bleeding tendency by impairing hemostasis [87,88,89,90], as seen in Figure 1. To this effect, we will focus on the evidence that classifies the proteins as activatory or negative and how this can trigger bleeding or thrombotic disorders.

For starters, the role of activatory signals within the cell is to amplify the stimulatory response coming from the outside. This signaling comprises every protein or factor in which the activatory state favors the platelet adhesion, aggregation, secretion, and/or shape change [90]. In this category, we can count proteins like Talin-1, Dok-1, or Rap1 that favor the activation of αIIbβ3, proteins that are associated with or are part of the cytoskeleton, like actin and kindlin [91], as well as other proteins that function downstream of G protein-coupled receptors (GPCRs), integrins or GPIb-IX-V that are relevant for secretion, aggregation or thrombus stabilization [92].

As a counterpart of the activatory pathways, there are endogenous negative signaling regulators that reduce or impede proper platelet function by blockage of the active site in activator proteins like CIB1 and M3BIM; other times, inactivation can be achieved by dephosphorylation by either the phosphatases like PP2B and PTEN, or even by using inhibitory compounds like CIB1 [93,94]. All of these break the positive feedback generated during the activation or maintain the quiescence inside the cells to control thrombus growth or bleeding time. These elements are helpful to diminish the hemostatic cascade and avoid spontaneous cardiovascular events [95,96].

Therefore, it is essential to understand the effect of each protein within the thrombi formation process and to see the impact of protein depletion, protein mutation, antibody-based inhibition, or pharmacological inhibition on bleeding time in vitro and in vivo.

Table 2 condenses the effects of protein depletion on hemostasis using protein inhibition or protein depletion experiments.

## 4. Proteins

As shown, the proteins in the platelet cannot be isolated from the overall process of activation or aggregation, as they could be downstream in several pathways and intervene in more than one process. To help understand each protein’s final contribution, they can be categorized based on whether they support or negatively regulate the activation process [95].

Activating proteins can be described as proteins that directly or indirectly contribute to activating various signaling cascades that lead to platelet shape change, aggregation, and secretion of granule contents. In contrast, negative regulatory proteins are those that prevent excessive or inappropriate platelet activation and thrombus formation, such as nitric oxide (NO) synthase, prostacyclin (PGI2) synthase, or phosphatases, which reverse activation and dampen the overall response.

In other words, proteins are clustered by their association with integrins, G proteins, secretion processes, or cytoskeleton dynamics.

### 4.1. Integrins and Integrin-Associated Proteins

Integrins are transmembrane proteins that act as receptors for various ligands, including collagen, fibronectin, and von Willebrand factor (vWF) and play a crucial role in platelet adhesion to injured vessel walls and other platelets [150]. Platelets express several integrins, the most critical being integrin αIIbβ3 (GPIIb/IIIa) [151]. This integrin exists in two main conformations: in its resting state, the integrin has a low affinity for its ligands, meaning it does not readily bind to other molecules, preventing unnecessary platelet activation in the absence of injury. Upon platelet activation by various stimuli (e.g., collagen exposure, thrombin binding), integrin αIIbβ3 undergoes a conformational change, dramatically increasing its affinity for ligands [152]. This “outside-in” signaling process is crucial for integrin function.

Integrin binding to ligands promotes adhesion and triggers additional signaling events within the platelet involving clustering, cytoskeletal reorganization, and secretion of activation molecules. However, this process is not exclusive to the integrin protein, as the regulation between resting and activated states depends on the integrin-associated proteins that act as adapters or co-activators for integrins. These proteins facilitate ligand binding and promote platelet activation and aggregation.

As expected, the lack of integrin αIIbβ1 decreases platelet adhesion to wounds, thus increasing the bleeding time by 3- to 4-fold. The same effect can be seen by another protein associated with integrin β, kindlin-3, whose deficiency impedes integrin β3 activation and is present in a rare genetic disorder called leukocyte adhesion deficiency type III (LAD-III) [97,153].

Dok proteins are a family of adaptor proteins; even though they are downstream of tyrosine kinase, their role in integrin assembly cannot be understated. It is suggested that Dok-1 is a negative regulator of αIIbβ3 “inside-out” signaling by working as an inhibitor of thrombosis in mice. Knock-out mice (Dok-1-/-) show a shortened bleeding time, clot retraction, PLCγ2 phosphorylation, and spreading, which are all signs of “outside-in” αIIbβ3 signaling [98].

Talin-1, another protein that mediates the linkage of integrin to the actin cytoskeleton, is necessary for its activation via integrin β1 and β3 [99,100] and is an essential biomarker for cancer [154], which facilitates platelet activation and thrombus formation, as mice with mutant L325R Talin-1 show increased bleeding times [104]. The small GTPase Rap1 is attached to this protein, which interacts with Talin-1 in the integrin activation pathway. The loss of interaction between Rap1 and Talin-1, achieved with the mutation of the interaction site of Talin-1 (R35E, R118E), resembles the effect of knock-out mice with an impedance in integrin-related activation [102].

Another protein in the same group is the integrin-linked kinase (ILK), an enzyme from the RAF subfamily, which has been associated with multiple cellular functions, including migration, proliferation, and adhesion, as its activity is linked to integrin β1 and β3 [103]. The lack of ILK in knockout mice is embryonic lethal because it lacks muscle development, and the inhibition or absence of functional ILK (conditional ILK deficient mice) reduces α-granule secretion, fibrinogen binding, thrombus stability, and an increase in bleeding time [104].

CD154 is a CD40 ligand that also binds integrin αIIbβ3, and its absence increases the bleeding time owing to defective hemostasis without changing the platelet count [105].

The protein ASK1, also known as MAP3K5, is a MAP kinase. The deletion of ASK1 results in lower αIIbβ3 activation and aggregation. It activates rapidly upon stimulation and protects against thromboembolism [99] without changing the TXA2 generation ratio [107].

ERp57, also known as PDIA3, is a member of the protein disulfide-isomerase (PDI) family of proteins that plays a crucial role in regulating cell growth and death in hypoxic environments. Recent studies have suggested ERp57 may also play a role in thrombus formation [110]. It has been proposed that ERp57 isomerase activity is required for proper fibrin generation [111], which is necessary for platelet aggregation and clot formation. In addition, ERp57 has been shown to interact with αIIbβ3 integrin, which is essential for platelet aggregation [155]. Studies have suggested that non-inhibitory antibodies against ERp57 may inhibit the function of αIIbβ3 integrin, leading to decreased platelet aggregation and thrombus formation. However, it is essential to note that the exact mechanism of action of non-inhibitory antibodies against ERp57 is not yet fully understood and may involve multiple pathways. Furthermore, non-inhibitory antibodies against ERp57 may affect platelet function beyond the inhibition of αIIbβ3 integrin [109]. Further research is needed to fully elucidate the role of ERp57 in thrombus formation and the potential therapeutic applications of targeting this protein.

Factor 3, or C3, is a protein in the complement system. It is helpful in innate immunity, especially for protection against bacterial infection; its deficiency, as shown by the increase in susceptibility to infections, decreases platelet activation and the frequency and size of thrombi [115].

Fyn is a tyrosine-protein kinase from the Src family, involved in several pathways like Par 1/4, αIIbβ3, glycoprotein VI (GPVI), GPIb-IX-V, and P2Y12; Fyn-/- mice, have shown many abnormalities in hemostasis with an increase in bleeding time [156]. CD148 is a tyrosine phosphatase whose absence in mice has shown reduced responsiveness and increased bleeding time [116].

Rap1b is a small GTPase, abundant in platelets and activated under GPCR stimulation; its absence in Rap1b-null platelets has a reduced response and longer bleeding times [157], contrary to its associated molecule RIAM, which shorter the bleeding time in vivo [158].

In addition, other proteins interact with integrin αIIbβ3, diminishing thrombus formation. One such protein is Dok-2, a scaffolding protein attached to αIIbβ3 that, contrary to Dok-1, can lessen the flux of calcium and PIP3 accumulation by being inhibited. This increases adhesion and activation, leading to faster thrombus formation in vivo and shorter bleeding time [134].

PP2B is a serine/threonine phosphatase dependent on calcium. The β isoform is associated with Filamin A and “outside-in” integrin signaling. The lack of PP2B-Aβ in mice caused a loss of dephosphorylation of Filamin A and decreased occlusion time in the artery [93].

GDF-15 is an inhibitory cytokine called MIC-1, which usually interferes with inflammation, cell growth, cell repair, and apoptosis. Due to increased pulmonary thromboembolism, the GDF-15-/- mice had rapid thrombus formation and a lower survival rate. These effects were reversed by incorporating recombinant GDF-15, reducing the sensibility to agonist and lower binding to fibrinogen; it is proposed that GDF-15 activates PKA and impedes “outside-in” αIIbβ3 activation [135].

PTPN11, known as SHP2, is a tyrosine phosphatase that regulates oncogenic transformation, differentiation, and cell growth. The SHP2-deficient thrombi are more stable and increase granule secretion without changes in response to agonists; it is proposed that Shp2 works downstream of integrin αIIbβ3 “outside-in” signaling, blocking dense granule secretion and AKT phosphorylation [136].

ADTM is an inhibitory compound targeting ERp57, which reduces arachidonic acid and ADP-induced aggregation, affecting P-selectin expression and αIIbβ3 integrin-dependent activation [137].

CIB1 is a known inhibitor associated with ASK1 in resting platelets with a low concentration of Ca^2+^; this protein detaches upon activation with thrombin. The lack of this inhibitor in CIB1-/- mice increased ASK1/p38 activation, suggesting its role as a negative regulator of platelet aggregation [138].

### 4.2. G Protein-Coupled Receptors (GPCRs)

GPCRs are transmembrane receptors that transduce signals from various stimuli, including thrombin and ADP, to activate internal signaling pathways, leading to platelet activation and aggregation.

Platelets use specific GPCRs on their surface for particular agonists, such as the ADP receptors (P2Y1 and P2Y12) or the Thrombin receptors (PAR1 and PAR4).

These proteins are associated with G proteins, which play a crucial role in platelet function and are essential for transmitting signals from the extracellular environment to the intracellular machinery. G proteins are heterotrimeric proteins composed of α, β, and γ subunits and are activated by GPCRs on the surface of platelets. Upon activation, the G protein α subunit dissociates from the βγ subunits and interacts with downstream effectors such as adenylyl cyclase or phospholipase C (PLC). Upon binding to an agonist, the GPCR undergoes a conformational change, triggering the activation of a G protein bound to its intracellular domain. G proteins are heterotrimeric, consisting of three subunits: α, β, and γ.

Upon activation, the α subunit dissociates from the βγ subunit and becomes GTP-bound (active), inhibiting downstream enzymes, such as phospholipase C (PLCβ). This leads to the production of inositol trisphosphate (IP3) and diacylglycerol (DAG). The βγ subunit directly interacts with downstream targets, activating kinases such as kinase C (PKC) and amplifying the signal.

The α-subunit of the G protein can be inhibitory (such as Gαi2) or stimulatory (such as Gαq), and its activity determines the intracellular signaling cascade that is activated. Gαi2 is an inhibitory subunit that inhibits adenylyl cyclase activity and reduces the intracellular levels of cyclic AMP (cAMP), increasing intracellular calcium levels. This increase in calcium level is essential for platelet activation and aggregation. The lack of Gαi2 can impair the ability of platelets to regulate intracellular calcium levels and reduce thrombus stability, leading to increased bleeding. Overall, the role of G proteins in platelet function is critical, and dysregulation of G protein signaling can lead to platelet disorders, such as thrombosis or bleeding disorders [122].

The role of G13 is crucial to the TXA2-dependent pathway, and the lack of this protein, but not of Gα12, decreases the activation and aggregation of TXA2, collagen, and thrombin. This sub-activation of the RhoA pathway explains the instability of thrombi in vivo, resulting in a severe hemostatic deficiency.

RIPK3 or RIP3 is a known receptor for its role in necrosis, apoptosis, and inflammatory cell death; however, new information has arisen regarding its role in hemostasis, and its interaction with Gα13 promotes activation and thrombosis with the use of knockout mice with prolonged bleeding times and reduced thrombus [123].

PKC is a family of proteins with at least ten isoforms divided into three subfamilies based on the use of Ca^2+^ and DAG. The main isoforms in platelets are α, β, δ, and θ, and another isoform called PKCε is present mainly in patients with acute myocardial infarction [129]. The double knockout of PKCθ and PKCε increases the bleeding time, showing the complementary role of both proteins in platelet activation [130].

Together with these G proteins, negative regulators of G proteins, such as RGS proteins, hinder signaling by enhancing the GTPase activity of the alpha subunit of the G protein, leading to the termination of the signaling cascade. RGS16 is a member of the RGS protein family that specifically interacts with the alpha subunits of the Gq and G13 proteins.

Studies have shown that RGS16 is essential in regulating platelet activation and hemostasis. In mice lacking RGS16, platelets are more sensitive to activation, leading to decreased bleeding time and increased susceptibility to thrombus formation in vivo. This suggests that RGS16 is an essential negative regulator of platelet activation and plays a critical role in maintaining hemostasis [142].

In this sense, IP3 and DAG generated by PLCβ activation act as second messengers, triggering a cascade of events within platelets, such as calcium mobilization, shape change, secretion of activation molecules, and increased integrin activity. To regulate the function of GPCRs, there are two proteins: the first comprises G protein-coupled receptor kinases (GRKs), which phosphorylate activated GPCRs, leading to their desensitization and eventual internalization, thereby stopping the signal; the second comprises GTPase-activating proteins (GAPs), which help convert active Gα subunits back to their inactive GDP-bound state, turning off the signal, and adaptors and signaling molecules, which relay signals from activated receptors to various downstream pathways, leading to platelet activation and aggregation. They include enzymes, kinases, and GTPases.

Proteins relevant to this process include the small GTPases Rap1 and RhoA [159]. The activation of these proteins has been shown to have a hemostatic effect, promoting platelet aggregation and clot formation [160]. The following are activatory proteins that affect bleeding time.

PDK1 is a master kinase, activated by PI3K, and is responsible for activating many kinases, such as Raf1 [113], AKT, protein kinase C (PKC), and others. Due to the role of PDK1 in calcium signaling within the platelet, which is crucial for the activation in collagen-dependent activation, PKD1-deficient mice showed long-term survival with lower ischemic stroke [112].

AKT1, commonly known as PKB, is a serine/threonine helpful kinase in many pathways, from cell survival and metabolism to platelet aggregation. As the interaction with most agonists triggers AKT activation, the lack of AKT in mice is expected to lead to defective granule release through calcium concentration [114].

iPLA2γ is a calcium-independent phospholipase relevant for maintaining lipid synthesis and homeostasis. In platelets, it is involved in a pro-thrombotic phenotype. Therefore, the lack of this protein diminishes the ADP-dependent aggregation and TXA2 production, with a longer bleeding time [124].

On the other hand, some proteins with a negative effect regulate the excessive activation of the platelet; one example is PTEN (Phosphatase and Tensin homolog), a phosphatase commonly known for its tumor-suppressive properties. PTEN functions by catalyzing the dephosphorylation of the signaling lipid PIP3, thereby regulating downstream signaling negatively. However, recent studies have shown that PTEN also plays a crucial role in regulating platelet function. In PTEN (-/-) mice, platelet count has been observed to increase by 25%, which shortens the bleeding time and increases platelet activation and aggregation in response to collagen. This effect on platelet function has been linked to the modulation of AKT phosphorylation, a downstream effector of the PI3K signaling pathway. The loss of PTEN in platelets leads to the activation of the PI3K–AKT pathway, resulting in increased platelet activation and aggregation. These findings highlight the role of PTEN in regulating platelet function and suggest that targeting PTEN signaling could have therapeutic implications for platelet-related disorders [139].

TRAF3 is a protein from the TNF receptor-associated factor family used as a mediator in the immune response of CD40. The signaling of CD40 in platelets induces aggregation independent from GPVI and integrin αIIbβ3, and the lack of TRAF3 increases this effect, as shown by the reduced bleeding time in knockout mice [143].

ELMO is a non-catalytic protein dedicated to cytokinesis, which increases the production of PIP3 by attachment to RhoG. The absence of this protein increases platelet aggregation, granule secretion, TXA2 generation, and integrin activation, with a lower bleeding time. The activity of RhoG increased without ELMO, suggesting a negative regulation of this pathway [140].

PECAM-1 or CD31 is an adhesion molecule related to integrin activation and angiogenesis; its deficiency increases platelet aggregability to vWF and the size of thrombi, suggesting a regulatory role of CD31 in the extent of platelet activation [161].

GSK3β is a multifunctional serine/threonine kinase susceptible to oxidative and ER stress. The lack of one allele in mice increases the sensibility to thrombosis, acting as a negative regulator in vivo [162].

Salidroside is a pharmacologically active glucoside of tyrosol extracted from Rhodiola rosea. It has anxiolytic and antidepressant effects in the mitochondria of dopaminergic neurons [141]; when injected, this compound increases the bleeding time and inhibits thrombus formation. It is suggested that the metabolic changes stimulate mitochondrial biogenesis and then affect the AKT/GSK3β signaling [141].

Glutamate receptor-interacting protein 1 (GRIP1) is a large scaffolding protein essential in organizing neurons’ proteins. GRIP1 interacts with the glycoprotein Ib-IX-V (GPIb-IX-V) receptor complex in platelets, critical for platelet adhesion and thrombus formation.

Studies using GRIP1-deficient mice have shown that the absence of GRIP1 does not affect platelet count but does impair platelet function. Specifically, the bleeding time in GRIP1-deficient mice is increased compared to wild-type mice, indicating impaired hemostasis. The interaction between GRIP1 and the GPIb-IX-V complex is essential for the proper localization and function of the GPIb-IX-V complex in platelets. The GPIb-IX-V complex binds von Willebrand factor (vWF) and mediates platelet adhesion to exposed subendothelial collagen at sites of vascular injury. In addition to its interaction with GPIb-IX-V, GRIP1 has also been shown to interact with other platelet proteins, such as the integrin αIIbβ3 and the G protein-coupled receptor PAR4, suggesting that it may play a broader role in platelet function [125].

TSSC6 is a protein from the tetraspanin superfamily. Its expression is restricted to hematopoietic cells and interacts specifically with the receptor P2Y12; TSSC6-deficient mice report smaller thrombosis, less hemostatic stability, and longer bleeding times [163].

Panx1 is a protein from the innexin family used as a structure for gap junctions, forming an anion-selective channel, helping the influx of Ca^2+^, ATP release, and platelet aggregation [126]. The Panx1-/- mice increase 2.5-fold the bleeding time, as well as a 2-fold platelet-specific deletion of Panx (Panx1PDel mice) [127].

ATP2A3 or SERCA3 is an intracellular pump in the endoplasmic reticulum that catalyzes ATP to translocate Ca^2+^ from the cytosol; SERCA3 knockout shows a longer bleeding time and defective adhesion to collagen [128].

### 4.3. Spreading and Cytoskeleton Dynamics

The pathway regulating platelet cytoskeleton dynamics is essential in hemostasis and thrombosis. Upon activation, these proteins trigger downstream effectors, which regulate various cytoskeletal proteins involved in actin–myosin interactions, which are necessary for platelet shape change, adhesion, and spreading [164].

Dysregulation of this pathway has been linked to platelet disorders, such as thrombosis and bleeding disorders, as well as cancer cell invasion and metastasis. Therefore, developing targeted therapies that modulate this process could have significant clinical implications for both platelet and cancer-related disorders.

FlnA or Filamin A is a protein that crosslinks actin filament to form the cytoskeleton; ubiquitous and widely expressed, it is known to interact with several proteins. The mutant “FlnA-null platelets” cannot rearrange their structure and spread, as α-granule secretion by integrin αIIbβ3 activation is impeded [117] and therefore affects the spreading process.

JNK1 is a kinase from the MAPK pathway downstream of MKK. Its role in thrombus formation and secretion was determined in JNK-/- mice, which had a 60% increase in bleeding time [118].

RhoA is a small GTPase protein that is crucial in regulating the clotting process. Studies have shown that RhoA-deficient platelets have reduced thrombotic responses and impaired clot retraction, indicating the essential role of RhoA in platelet function. Additionally, RhoA signaling has been linked to generating reactive oxygen species (ROS) in platelets, which can contribute to thrombosis. Even though RhoA signaling could be a potential therapeutic strategy for preventing and treating thrombotic disorders [119], such treatments must consider the associated risks.

ROCK2 is a serine/threonine kinase downstream of RhoA and plays a crucial role in regulating the formation of actin fibers. In contrast to other proteins that interact with actin, ROCK2 has been found to have a specific role in thrombus formation, particularly in stabilizing thrombi. Studies have shown that ROCK2-deficient platelets (ROCK2Plt-/-) form thrombi with lower stability than wild-type platelets, indicating the critical role of ROCK2 in thrombus stability. However, interestingly, when the response to pre-formed clots was tested, ROCK2Plt-/- mice showed a similar reaction to wild-type mice, suggesting that ROCK2 may only be essential for the initial formation of a clot. These findings highlight the complex and dynamic nature of platelet function and the need for further research to fully understand the role of ROCK2 in thrombus formation and stability [121].

IKKβ is an enzymatic component of the cytokine-activated intracellular signaling pathway; the phosphorylation of the inhibitor of NF-Kβ (IKβ) and subsequent release of NF-Kβ. This increase in NF-Kβ also increases platelet aggregation along with several other physiological processes, and therefore, the lack of IKKβ is fatal for the embryo. Platelet-specific deficiency of IKKβ does affect arterial neointima formation and platelet spreading [133].

### 4.4. Platelet Secretion

Along with platelet spreading, granule secretion is a vital process associated with initiation of the activation and aggregation of neighboring platelets.

One critical effector in the granule secretion process is VPS33B, a vacuolar protein used for docking/fusion of endosomes and lysosomes; the lack of this protein in knockout mice reduced the vesicular trafficking and impeded the α-granule biogenesis [120].

Munc-18 proteins are essential components of the synaptic vesicle fusion protein complex required for the exocytosis of granules. The ablation of their second paralog impedes the release of alpha, dense, and lysosomal granules with a severe hemostatic defect and longer bleeding times [131]. 

HMGB1 or high mobility group boxes are nuclear proteins that bind to DNA, but analysis of isolated platelets showed an upregulation in patients with acute ischemic events; the HMGB1-/- mice had reduced platelet aggregation, inflammation, and thrombus formation, revealing an essential role in granule secretion, adhesion, and spreading [132].

This process can also be negatively regulated by TMX1, a transmembrane disulfide isomerase from the same family as ERp57 or PDI, with an increased expression in thrombin-stimulated platelets; this negative feedback was confirmed using an anti-TMX1 antibody and a TMX1 knockout, both of which exhibited an increase in ATP release, P-selectin expression, and larger thrombi size [145].

CLP36 or PDLIM1 is a negative regulator of GPVI, impeding GPVI-mediated signaling and the consequent platelet activation. The lack of PDLIM1, as seen in CLP36-/- mice, increases the response to GPVI agonist by increasing Ca^2+^ and granule secretion, resulting in a prothrombotic phenotype [144].

## 5. Inhibitory Compounds with Effect on the Bleeding Time

M3BIM is a synthetic benzimidazole-derived oligosaccharide reported in 2016 as inhibitory of the platelet activation induced by thrombin and collagen without toxicity by inhibiting the phosphorylation of phospholipase Cγ2 and the MAPKs; its activity slightly increases the bleeding time in mice [146].

Palladin is a protein that regulates the actin cytoskeleton in motility, embryonic development, and cytoskeletal organization. Within platelets, the lack of one allele of paladin decreases the bleeding time and the occlusion time by 30 percent [147].

Timosaponin AIII is an inhibitor of the TP receptor that affects PKC; its inhibition was not dependent on cAMP, cGMP, or TXA2 production. This compound increases bleeding time and reduces thrombus size, as does thromboembolism in general [148].

DCa, DHEA, and DASA are other compounds that increase bleeding time in mice. These compounds affect aerobic glycolysis and the pentose phosphate pathway [149].

## 6. Proteins without a Significative Difference in Bleeding Time or Thrombus Formation

Some proteins are involved in the aggregation, activation, or coagulation cascade, but their absence does not meaningfully affect the bleeding time; these proteins are not to be mistaken as risk-free targets.

PKCι/λ is an atypical isoform that requires phosphatidyl serine instead of Ca^2+^ or DAG for activation; its function comes from the microtubule dynamics tasked with secretion. As shown by knockout mice [165], its association with CDC42 did not affect hemostasis or thrombosis but has been shown to impair glucose transport in muscle [166].

IKK2 knockout platelets showed no functional impairment in vivo or in vitro. Bleeding time and thrombus formation were not affected in platelet-specific IKK2-knockout mice. Moreover, platelet aggregation, glycoprotein GPIIb/IIIa activation, and degranulation were unaltered. These observations were confirmed by pharmacological inhibition of IKK2 with TPCa-1 and BMS-345541, which did not affect the activation of murine or human platelets over a wide concentration range. Results imply that IKK2 is not essential for platelet function [167] but did elevate interleukin-6, neutrophilia, and fatal gastrointestinal inflammation [168].

The microtubule-actin cross-linking factor 1 (MACF1; synonym: Actin cross-linking factor 7, ACF7) is a member of the spectraplakin family and one of the few proteins expressed in platelets, which possesses actin and microtubule-binding domains thereby facilitating actin-microtubule interaction and regulation. MACF1 deficient mice displayed comparable platelet counts to control mice. The platelet cytoskeletal ultrastructure analysis revealed a regular marginal band and actin network. Platelet spreading on fibrinogen was slightly delayed, but platelet activation and clot traction were unaffected. Ex vivo thrombus formation and mouse tail bleeding responses were similar between control and mutant mice [169]. Nevertheless, this knockout model showed developmental retardation and embryonic lethality [170].

YM-254890 is a specific Gq and G11 inhibitor that decreases the intracellular Ca^2+^ with an IC_50_ of 0.9^2+^/−0.28 µM. Its effect decreases the thrombus formation without prolonging bleeding time [171].

BAMBI is a transmembrane protein related to the TGFB superfamily and is highly expressed in platelets and endothelial cells; this protein is different from the latter because it did not affect platelets activation, procoagulant function or platelet counts in knockout mice but instead increased the bleeding time and instability of thrombi by damaging the endothelium instead of platelets [172] and rising ROS [173].

## 7. Clinical Relevance

After revisiting the biological basis of protein inhibition at various stages, we must focus on the potential applications, clinical significance, and how these findings might evolve.

Advances in personalized antiplatelet therapy show that while aspirin remains a valuable treatment, it is not entirely sufficient, with a recurrence rate of 827 out of 10,722 strokes. Dual antiplatelet therapy can lower this recurrence by 29%, but it also more than doubles the risk of bleeding [174].

Additionally, the risk of thrombosis is higher in women, who tend to experience a greater incidence of major cardiovascular and cerebrovascular events when using clopidogrel, alongside bleeding risks associated with other P2Y12 inhibitors [175]. Age further complicates platelet reactivity, especially with co-morbidities such as frailty, hypertension, or diabetes, which can exacerbate inflammation, alter cellular metabolism, and impact other physiological processes.

Given the variability in platelet proteins and how they may influence treatment outcomes, there is an urgent need for comprehensive proteomic studies across diverse patient populations and disease stages. Such research could lead to treatments tailored not just to the symptoms but also to the underlying pathophysiology of the disease.

This knowledge is also vital in the development of targeted drugs. In the quest for new therapeutic targets for conditions like cancer, hypertension, or metabolic diseases, off-target inhibition can inadvertently cause increased bleeding or a higher risk of thrombosis. For example, the integrin-related protein Talin-1 has been strongly linked with the invasive and migratory processes driving metastasis [176]. Therefore, drugs aimed at inhibiting this protein or others in the same pathway could theoretically increase bleeding risks [177].

Just as measuring Amyloid Precursor Protein (APP) levels in platelets can serve as a diagnostic tool for assessing Alzheimer’s disease severity [178], it is reasonable to consider protein levels as biomarkers for future complications. This is evident in using RNA sequencing to identify platelet hyperreactivity [179].

On another note, many promising compounds—from plant extracts to nitrated lipids or peptides developed as antiplatelet agents—might increase bleeding when tested in vivo. This is not uncommon. Understanding the potential effects of inhibiting specific proteins early on can eliminate such compounds in the initial screening stages, streamlining drug development and reducing the risk of adverse outcomes.

Similarly, problems can arise when a protein essential for activating an orally administered drug is not metabolized correctly due to issues with activating specific proteins [180].

## 8. Future of Platelet Signaling and the Pathway Forward

**Figure 2:**
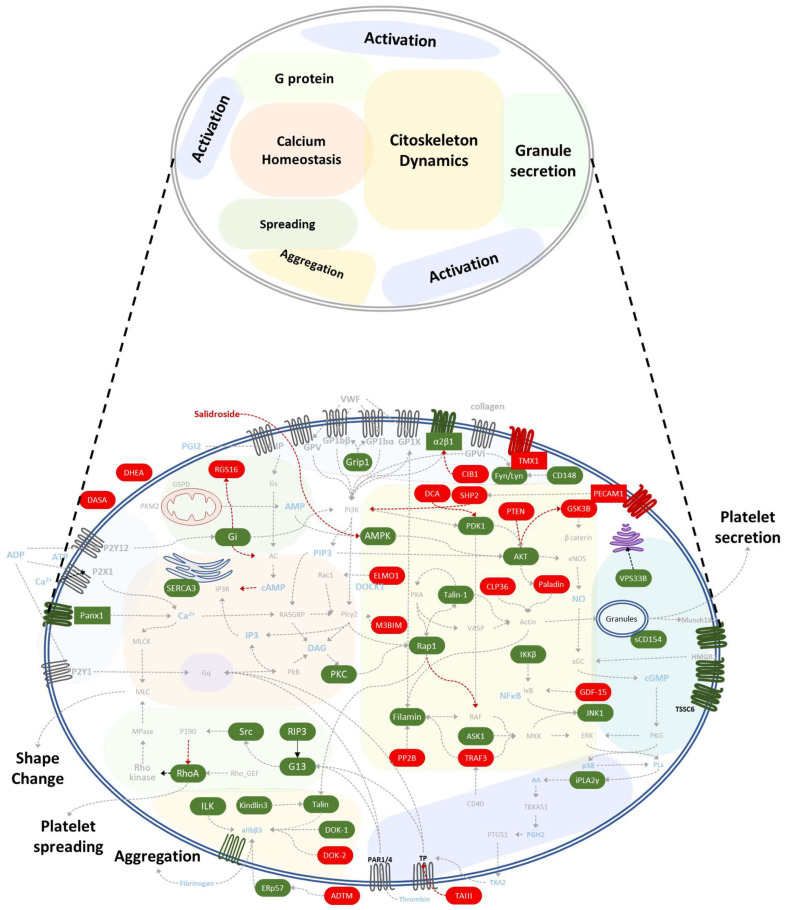
Platelet signaling pathways are interconnected. The platelet signaling pathway is a complex network of molecules and events involved in platelet activation, secretion, shape change, aggregation, and calcium homeostasis. Proteins are rectangles, inhibitors are circles, transmembrane proteins are hexagons, and compounds are words without borders. The green background stands for activatory proteins, while the red background is for proteins from negative signaling, the red arrow represents inhibition, and the grey arrow represents normal interaction. AKT: Protein Kinase B; ASK1: Apoptosis Signal-regulating Kinase 1; C3: Complement component 3; Ca^2+^: Calcium; CD148: protein tyrosine phosphatase receptor type J; CD154: CD40 ligand; CD40: Tumor necrosis factor receptor superfamily member 5; CIB1: Calcium and integrin-binding protein 1; CLP36: PDZ and LIM domain protein 1; DASA: diarylsulfonamide; DCa: dichloroacetate; DHEA: dehydroepiandrosterone sulphate; Dok-1: Docking protein 1; Dok-2: Docking protein 2; ELMO1: Engulfment and cell motility protein 1; ERK: Mitogen-activated protein kinase 15; ERp57: Protein disulfide-isomerase A3; FlnA: Filamin A; Fyn: Tyrosine-protein kinase Fyn; GDF-15: Growth/differentiation factor 15; Gi: Guanine nucleotide-binding protein G(i); GPCRs: G protein-coupled receptor; GPIb: Platelet glycoprotein Ib; GPV: Platelet glycoprotein V; GPIX: Platelet glycoprotein IX; GPVI: Platelet glycoprotein VI; GRIP1: Glutamate receptor-interacting protein 1; GSK3β: Glycogen synthase kinase-3 beta; Gα(i2): Guanine nucleotide-binding protein G(i2) subunit alpha; Gα13: Guanine nucleotide-binding protein G(13) subunit alpha-1; HMGB1: High mobility group protein B1; IKKβ: Inhibitor of nuclear factor kappa-B kinase subunit beta; ILK: Integrin-linked protein kinase; iPLA2γ: Calcium-independent phospholipase A2-gamma; IκB: NF-kappa-B inhibitor alpha; JNK1: Mitogen-activated protein kinase 8; KIND3: Fermitin family homolog 3; M3BIM: benzimidazole-derived oligosaccharide; MEK: Mitogen-activated protein kinase kinase; Munc18-2: Mammalian Uncoordinated-18; P2X1: P2X purinoceptor 1; P2Y12: P2Y purinoceptor 12; p38 MAPK: Mitogen-activated protein kinase 11; Panx: Pannexin-1; PAR1: Protease-activated receptor-1; PDK1: 3-Phosphoinositide-dependent protein kinase 1; PECAM-1: Platelet and Endothelial Cell Adhesion Molecule 1; PI3K: Phosphatidylinositol 4,5-bisphosphate 3-kinase; PKA: protein kinase A; PKC: protein kinase C; PP2B: Serine/threonine-protein phosphatase 2B; PTEN: Phosphatase and tensin homolog; RAC1: Ras-related C3 botulinum toxin substrate 1; raf1: proto-oncogene c-RAF; Rap1: Ras-related protein 1; RGS 16: Regulator of G protein signaling 16; RhoA: Ras homolog family member A; RhoG: Ras homolog family member G; RIAM: Rap1–GTP-interacting adapter molecule; RIP3: Receptor-interacting protein kinase 3; ROCK: Rho-associated protein kinase; SERCA3: Sarcoplasmic/endoplasmic reticulum Calcium ATPase 3; SFK: Src kinase family; Shp2: Src homology region 2-containing protein tyrosine phosphatase 2; SNARE: SNAP Receptors; Src: Proto-oncogene tyrosine-protein kinase Src; TAIII: timosaponin A-III; TLR4/cGKI: toll-like receptor 4; TMX1: Thioredoxin-related Transmembrane Protein 1; TNFR: Tumor necrosis factor receptors; TP receptor: thromboxane receptor; TRAF3: TNF receptor-associated factor 3; TSSC6: Tumor-suppressing STF cDNA 6; TXA2: thromboxane A2; VPS33B: Vacuolar protein sorting-associated protein 33B.

The inner workings of platelets have garnered increasing attention in recent years, primarily due to their involvement in a wide range of pathological processes, from inflammation to cancer. The complexity of platelet biology has revealed a highly interconnected network of signaling pathways (Figure 2), making it difficult to pinpoint the final effect of any specific protein by merely following a downstream pathway. This intricate web of interactions within platelet signaling highlights the need for a more nuanced understanding of how these pathways contribute to various diseases.

Despite significant progress, much remains to be discovered about the internal mechanisms of platelets. This knowledge gap underscores the necessity for developing and applying innovative technological approaches. Traditional methods, such as searching for new agonists [181], continue to be valuable, but emerging technologies are opening new avenues for research. The study of microRNAs [182], lipidomics [183,184], and other multi-omics approaches [185], combined with bioinformatic analyses [186,187,188], have enabled the integration of vast clinical datasets. This interdisciplinary approach is essential for unraveling the complex mechanisms governing platelet function and their role in disease progression. The large datasets generated, coupled with the use of AI and Big Data, allow us to better understand each patient’s pathology and medical needs [189,190].

Furthermore, as new and particular compounds targeting emerging biological targets become more common, it is crucial to consider the broader implications of these interventions. Even when a compound is designed with a single protein target in mind, off-target effects can become severe, as seen with chemotherapeutic compounds that increase the risk of thrombosis [191,192].

Once these complications are identified, they can be addressed—not with aspirin but by reversing the effect downstream in the same pathway. This approach anticipates and mitigates potential adverse effects in developing new therapeutic agents.

## 9. Future Considerations

As research into blood-related disorders advances, platelets are increasingly recognized as pivotal players in inflammation and hemostasis [10,193]. Despite the significant progress made, our understanding of platelet biology remains incomplete. With each discovery, the complexity of this field only deepens [194]. This complexity presents challenges and opportunities: while it complicates the study of platelet function, it also opens the door to innovative approaches for more accurately describing and manipulating platelet activity [195,196].

One major limitation in our current understanding is the lack of comprehensive insight into how individual proteins within platelet signaling pathways interact and contribute to overall platelet function. Although advances in genetic modulation technologies have enabled researchers to modify specific proteins selectively, the full impact of these modifications on platelet behavior and disease processes is not yet fully understood [197]. This gap in knowledge underscores the need for more in-depth studies to elucidate the downstream effects of genetic alterations, especially in the context of complex diseases like cancer and inflammatory disorders, where platelets play a critical role [198].

Looking forward, future research should prioritize the development of more sophisticated models and tools to study platelet biology in greater detail. Integrating multi-omics approaches combining genomics, proteomics, and metabolomics could offer a more holistic understanding of platelet function and its role in various diseases. Additionally, there is an urgent need for improved in vitro and in vivo models that more accurately mimic the physiological environment of platelets, enabling better predictions of therapeutic outcomes. In the context of venous thromboembolism (VTE) in metabolic diseases such as diabetes and cancer [199], it is crucial to develop strategies that effectively reduce the risk of thrombosis while minimizing the associated bleeding risks. Striking this delicate balance remains one of the most significant challenges in antiplatelet therapy [200].

Moreover, as new therapies targeting platelet function are developed, it is essential to consider their long-term effects and potential off-target impacts. This can be achieved by increasing the scope of longitudinal studies or by analyzing clinical data from patients with diverse backgrounds, pathologies, co-morbidities, and treatments. A deeper understanding of the variability and molecular mechanisms driving platelet-related pathologies will be critical in refining these therapies to maximize efficacy while minimizing adverse outcomes.

Future research must also focus on personalized approaches that account for patients’ genetic and proteomic variability, allowing for treatments tailored to individual risks and disease profiles. By addressing these challenges, the field can advance toward more effective and safer therapeutic interventions, ultimately improving patient outcomes in conditions where platelet function is a crucial factor.

## Figures and Tables

**Table 1:** Diseases related to pathway mutation or failure.

Pathway	Role	Diseases	References
Granule secretion	This pathway contemplates proteins used for cytoskeletal organization, intracellular calcium levels, kinase activity, and intracellular protease activity, which facilitate membrane fusion and granule secretion from platelets.	Stroke, cerebral infarction, chronic inflammatory bowel disease, sepsis, lupus, atherosclerosis, acute malaria, T-cell dysfunction in HIV-1.	[14,15,16,17]
Integrin	Integrins are receptors in the membrane that mediate signals in response to the extracellular matrix, such as motility, cell shape, and cell cycle progression.	Thrombosis, coronary artery disease, inflammatory bowel disease, glioblastoma, dry eye disease, cancer, fibrosis, respiratory and neurological diseases, multiple sclerosis, SARS-Cov-2 infection, diabetic retinopathy, glaucoma, optic nerve degeneration, Alzheimer’s disease, Crohn’s disease, nephropathy, and asthma.	[18,19,20,21,22,23,24,25,26,27,28,29,30]
GPCRs pathway	GPCR binds external molecules, activating G proteins and activating or inhibiting pathways for CaMP signaling and phosphatidylinositol signaling.	Retinitis pigmentosa, nephrogenic diabetes insipidus, obesity, heart failure, asthma, hypo- and hyperthyroidism, fertility disorders, and carcinoma.	[31,32,33,34,35,36,37,38,39,40,41,42,43,44,45,46]
MAPKs pathway	The pathway of MAPK/ERK transduces signals downstream of cytokine receptors and regulates the transcription of specific proteins and the integrin outside-in pathway.	Alzheimer’s disease, Parkinson’s disease, amyotrophic lateral sclerosis, cancer, and lymphoma.	[47,48,49,50,51,52,53,54,55,56,57,58,59,60]
Rho/ROCK pathway	The ROCK pathway involves the use of serine–threonine protein kinase in cell movement and reorganization of the cytoskeleton.	Alzheimer’s disease, Parkinson’s disease, ischemic myocardial fibrosis, atherosclerosis, restenosis, hypertension, pulmonary hypertension, cardiac hypertrophy, amyotrophic lateral sclerosis, and cancer.	[61,62,63,64,65,66,67,68,69,70,71]
Tyrosine kinase pathways	Tyrosine kinases are cell-surface proteins that act as signal transducers, regulating cellular processes such as apoptosis, proliferation, metabolism, and differentiation.	Cancer, vascular diseases, fibrosis, immune-mediated disorders, rheumatoid arthritis, bone disorders, arteriosclerosis, Alzheimer’s disease, and Parkinson’s disease.	[72,73,74,75,76,77,78,79]
Thromboxane pathway	Thromboxane synthesis induces pro-inflammatory and pro-thrombotic phenotypes by metabolizing PUFA from the membrane.	Atherosclerosis, hypertension, diabetes, obesity, myocardial infarction, and stroke.	[80,81,82,83,84,85]

**Table 2:** Proteins are related to either an increase or decrease in bleeding risk and associated depletion experiments.

Protein	Role	Type of Protein	Pathway Involved	Target	Depletion Experiment	Reference
Integrin αIIβ1	Activatory	Integrin	Integrin αIIβ1		αIIβ1-/-	[97]
Integrin αIIbβ3	Activatory	Integrin	Integrin αIIbβ3		αIIbβ3-/-	[97]
Kindlin-3	Activatory	Signaling protein	Integrin αIIbβ3	Talin	KIND3-/-	[97]
Dok-1	Activatory	adapter protein	Integrin αIIbβ3	integrin αIIbβ3	Dok-1-/-	[98]
Talin-1	Activatory	Cytoskeletal protein	Integrin αIIbβ3	integrin β1 and β3	Talin-1 (L325R)	[99,100,101]
Rap1	Activatory	Gtpase	Integrin αIIbβ3	Talin-1	Rap1-/-	[102]
ILK	Activatory	Kinase	Integrin αIIbβ3	integrin β1 and β3	ILK-/-	[103,104]
CD154	Activatory	Ligand of CD40	Integrin αIIbβ3	CD40	CD154-/-	[105]
ASK1	Activatory	MAP kinase	Integrin αIIbβ3	p38 MAPK	ASK1-/-	[106,107]
ERp57	Activatory	Disulfide isomerase	Integrin αIIbβ3	integrin αIIbβ3	ERp57-/- anti-ERp57	[108,109,110,111]
PDK1	Activatory	Kinase	P2Y12	raf1	PDK1-/-	[112,113]
AKT1	Activatory	Serine/threonine-protein kinase	AKT/GSK3β	GSK3β	AKT1-/-	[114]
Factor 3 (C3)	Activatory	Cofactor	Complement	Rap1b	C3-/-	[115]
Fyn	Activatory	Src family	Integrin αIIbβ3	Panx1	Fyn-/-	
CD148	Activatory	Protein Tyrosine Phosphatase	GPVI and SFK	GPVI	CD148-/-	[116]
Filamin	Activatory	Actin-binding protein	Spreading and granule release	Actin	FlnA-/-	[117]
JNK1	Activatory	MAP kinase	MEK/ERK	PKC	JNK1-/-	[118]
RhoA	Activatory	GTPase	RhoA-ROCK-MLC	ROCK	RhoA-/-	[119]
VPS33B	Activatory	Sorting protein	RhoA-ROCK-MLC	integrin	VPS33B-/-	[120]
ROCK	Activatory	Serine/threonine kinase	RhoA-ROCK-MLC	Actin	ROCK2Plt-/-	[121]
Gα(i2)	Activatory	G protein	GPCRs	Adenylate cyclase	Gα(i2)-/-	[122]
Gα13	Activatory	G protein	GPCRs	RhoA	Gα13-/-	
RIP3	Activatory	serine/threonine kinase	GPCRs	Gα13	RIP3 inhibitor	[123]
Rap1b	Activatory	GTPase	GPCRs	RIAMS preading	Rap1b-/-	
iPLA2γ	Activatory	Phospholipase	TXA2 production	Fatty acids	iPLA2γ-/-	[124]
GRIP1	Activatory	Receptor interacting protein	GPIb-IX, GPIbα, GPIbβ, GPIb-IX	GPIb-IX, GPIbα, GPIbβ, GPIb-IX	GRIP1-/-	[125]
TSSC6	Activatory	Integral membrane protein	Integrin	integrin αIIbβ3	TSSC6-/-	
Panx1	Activatory	ATP channel	integrin αIIbβ3	P2X1	Panx-/- PanxPDEL BrilliantBlue-FCF	[126,127]
SERCA3	Activatory	Intracelular Ca^2+^ pump ATPase	Granule secretion	Ca^2+^	SERCA3-/-	[128]
PKC	Activatory	Kinase	GPVI and MEK/ERK	PAR1, ERK	PKCθ-/- and PKCε-/-	[129,130]
Munc18-2	Activatory	Vesicle docking protein	Granule secretion	SNARE	Munc18-2-/-	[131]
HMGB1	Activatory	DNA binding protein	TLR4/cGKI	TLR4/cGKI	HMGB1-/-	[132]
IKKβ	Activatory	IκB kinase	Integrin αIIbβ3	IκB	IKKβ-/-	[133]
Dok-2	Negative	Adapter protein	Tyrosine kinase	integrin αIIbβ3	Dok-2-/-	[134]
PP2B	Negative	Phosphatase	Src	Filamin A	PP2B-Aβ-/- PP2B-Aβ depleted	[93]
GDF-15	Negative	Growth factor	integrin αIIbβ3 and αIIβ1	PKA	GDF-15-/-	[135]
Shp2	Negative	Tyrosine phosphatase	integrin αIIbβ3 and αIIβ1	AKT	Shp2-/-	[136]
ADTM	Inhibitory	Compound	integrin αIIbβ3	ERp57		[137]
CIB1	Negative	Calcium and integrin binding	integrin αIIbβ3	ASK1	CIB1-/-	[138]
PTEN	Negative	Phosphatase	AKT/GSK3β	PI3K	PTEN-/-	[139]
ELMO1	Negative	RAC1 activator	RhoG	RAC1	ELMO1-/-	[140]
PECAM-1	Negative	Transmembrane glycoprotein	GPIb/V/IX integrin AKT/GSK3β	PI3K	PECAM-1-/-	
GSK3β	Negative	Kinase	AKT/GSK3β	b Catenin	GSK3β-/-	
Salidroside	Inhibitory	Compound	AKT/GSK3β	Mitochondria		[141]
RGS 16	Negative	G protein regulator	GPCRs	Gi	RGS-16-/-	[142]
TRAF3	Negative	TNFR-associated factor	Integrin αIIbβ3	CD40	TRAF3-/-	[143]
CLP36	Negative	Cytoskeleton-associated protein	GPVI	GPVI	CLP36-/- CLP36 (ΔLIM)	[144]
TMX1	Negative	Transmembrane protein	integrin αIIbβ3	integrin αIIbβ3	TMX1-/-	[145]
M3BIM	Inhibitory	Compound	PLC/PKC/MAPKs	PKC		[146]
palladin	Negative	Actin-associated protein	Spreading	RAC1	palladin-/+	[147]
TAIII	Inhibitory	Compound	TP receptor	PKC		[148]
DCa	Inhibitory	Compound	AKT/GSK3β	Mitochondria		[149]
DHEA	Inhibitory	Compound	AKT/GSK3β	Mitochondria		[149]
DASA	Inhibitory	Compound	AKT/GSK3β	Mitochondria		[149]

AKT: Protein Kinase B; ASK1: Apoptosis Signal-regulating Kinase 1; C3: Complement component 3; Ca^2+^: Calcium; CD148: protein tyrosine phosphatase receptor type J; CD154: CD40 ligand; CD40: Tumor necrosis factor receptor superfamily member 5; CIB1: Calcium and integrin-binding protein 1; CLP36: PDZ and LIM domain protein 1; DASA: diarylsulfonamide; DCa: dichloroacetate; DHEA: dehydroepiandrosterone sulphate; Dok-1: Docking protein 1; Dok-2: Docking protein 2; ELMO1: Engulfment and cell motility protein 1; ERK: Mitogen-activated protein kinase 15; ERp57: Protein disulfide-isomerase A3; FlnA: Filamin A; Fyn: Tyrosine-protein kinase Fyn; GDF-15: Growth/differentiation factor 15; Gi: Guanine nucleotide-binding protein G(i); GPCRs: G protein-coupled receptor; GPIb: Platelet glycoprotein Ib; GPV: Platelet glycoprotein V; GPIX: Platelet glycoprotein IX; GPVI: Platelet glycoprotein VI; GRIP1: Glutamate receptor-interacting protein 1; GSK3β: Glycogen synthase kinase-3 beta; Gα(i2): Guanine nucleotide-binding protein G(i2) subunit alpha; Gα13: Guanine nucleotide-binding protein G(13) subunit alpha-1; HMGB1: High mobility group protein B1; IKKβ: Inhibitor of nuclear factor kappa-B kinase subunit beta; ILK: Integrin-linked protein kinase; iPLA2γ: Calcium-independent phospholipase A2-gamma; IκB: NF-kappa-B inhibitor alpha; JNK1: Mitogen-activated protein kinase 8; KIND3: Fermitin family homolog 3; M3BIM: benzimidazole-derived oligosaccharide; MEK: Mitogen-activated protein kinase kinase; Munc18-2: Mammalian Uncoordinated-18; P2X1: P2X purinoceptor 1; P2Y12: P2Y purinoceptor 12; p38 MAPK: Mitogen-activated protein kinase 11; Panx: Pannexin-1; PAR1: Protease-activated receptor-1; PDK1: 3-Phosphoinositide-dependent protein kinase 1; PECAM-1: Platelet and Endothelial Cell Adhesion Molecule 1; PI3K: Phosphatidylinositol 4,5-bisphosphate 3-kinase; PKA: protein kinase A; PKC: protein kinase C; PP2B: Serine/threonine-protein phosphatase 2B; PTEN: Phosphatase and tensin homolog; RAC1: Ras-related C3 botulinum toxin substrate 1; raf1: proto-oncogene c-RAF; Rap1: Ras-related protein 1; RGS 16: Regulator of G protein signaling 16; RhoA: Ras homolog family member A; RhoG: Ras homolog family member G; RIAM: Rap1–GTP-interacting adapter molecule; RIP3: Receptor-interacting protein kinase 3; ROCK: Rho-associated protein kinase; SERCA3: Sarcoplasmic/endoplasmic reticulum Calcium ATPase 3; SFK: Src kinase family; Shp2: Src homology region 2-containing protein tyrosine phosphatase 2; SNARE: SNAP Receptors; Src: Proto-oncogene tyrosine-protein kinase Src; TAIII: timosaponin A-III; TLR4/cGKI: toll-like receptor 4; TMX1: Thioredoxin-related Transmembrane Protein 1; TNFR: Tumor necrosis factor receptors; TP receptor: thromboxane receptor; TRAF3: TNF receptor-associated factor 3; TSSC6: Tumor-suppressing STF cDNA 6; TXA2: thromboxane A2; VPS33B: Vacuolar protein sorting-associated protein 33B.

## Data Availability

No new data were created or analyzed in this study. Data sharing is not applicable to this article.

## Glossary

AKT	Protein Kinase B
ASK1	Apoptosis Signal-regulating Kinase 1
C3	Complement component 3
Ca^2+^	Calcium
CD148	Protein tyrosine phosphatase receptor type J
CD154	CD40 ligand
CD40	Tumor necrosis factor receptor superfamily member 5
CIB1	Calcium and integrin-binding protein 1
CLP36	PDZ and LIM domain protein 1
DASA	Diarylsulfonamide
DCa	Dichloroacetate
DHEA	Dehydroepiandrosterone sulphate
Dok-1	Docking protein 1
Dok-2	Docking protein 2
ELMO1	Engulfment and cell motility protein 1
ERK	Mitogen-activated protein kinase 15
ERp57	Protein disulfide-isomerase A3
FlnA	Filamin A
Fyn	Tyrosine-protein kinase Fyn
GDF-15	Growth/differentiation factor 15
Gi	Guanine nucleotide-binding protein G(i)
GPCRs	G protein-coupled receptors
GPIb	Platelet glycoprotein Ib
GPV	Platelet glycoprotein V
GPIX	Platelet glycoprotein IX
GPVI	Platelet glycoprotein VI
GRIP1	Glutamate receptor-interacting protein 1
GSK3β	Glycogen synthase kinase-3 beta
Gα(i2)	Guanine nucleotide-binding protein G(i2) subunit alpha
Gα13	Guanine nucleotide-binding protein G(13) subunit alpha-1
HMGB1	High mobility group protein B1
IKKβ	Inhibitor of nuclear factor kappa-B kinase subunit beta
ILK	Integrin-linked protein kinase
iPLA2γ	Calcium-independent phospholipase A2-gamma
IκB	NF-kappa-B inhibitor alpha
JNK1	Mitogen-activated protein kinase 8
KIND3	Fermitin family homolog 3
M3BIM	Benzimidazole-derived oligosaccharide
MEK	Mitogen-activated protein kinase kinase
Munc18-2	Mammalian Uncoordinated-18
P2X1	P2X purinoceptor 1
P2Y12	P2Y purinoceptor 12
p38 MAPK	Mitogen-activated protein kinase 11
Panx	Pannexin-1
PAR1	Protease-activated receptor-1
PDK1	3-Phosphoinositide-dependent protein kinase 1
PECAM-1	Platelet and Endothelial Cell Adhesion Molecule 1
PI3K	Phosphatidylinositol 45-bisphosphate 3-kinase
PKA	Protein kinase A
PKC	Protein kinase C
PP2B	Serine/threonine-protein phosphatase 2B
PTEN	Phosphatase and tensin homolog
RAC1	Ras-related C3 botulinum toxin substrate 1
Raf1	Proto-oncogene c-RAF
Rap1	Ras-related protein 1
RGS 16	Regulator of G protein signaling 16
RhoA	Ras homolog family member A
RhoG	Ras homolog family member G
RIAM	Rap1–GTP-interacting adapter molecule
RIP3	Receptor-interacting protein kinase 3
ROCK	Rho-associated protein kinase
SERCA3	Sarcoplasmic/endoplasmic reticulum Calcium ATPase 3
SFK	Src kinase family
Shp2	Src homology region 2-containing protein tyrosine phosphatase 2
SNARE	SNAP receptors
Src	Proto-oncogene tyrosine-protein kinase Src
TAIII	Timosaponin A-III
TLR4/cGKI	Toll-like receptor 4
TMX1	Thioredoxin-related Transmembrane Protein 1
TNFR	Tumor necrosis factor receptors
TP receptor	Thromboxane receptor
TRAF3	TNF receptor-associated factor 3
TSSC6	Tumor-suppressing STF cDNA 6
TXA2	Thromboxane A2
VPS33B	Vacuolar protein sorting-associated protein 33B
